# Epstein-Barr virus infection-associated cholangiocarcinoma: a report of one case and the review of literature

**DOI:** 10.1186/s12985-022-01862-7

**Published:** 2022-08-09

**Authors:** Xinchun Zhang, Ning Wang, Wenyan Wei, Yangsheng Li

**Affiliations:** 1 Department of Radiology, Hangzhou Tianshui Wulin Street Community Health Service Centers, Hangzhou, 310006 China; 2 Department of Medical Examination Center, Affiliated Hangzhou First People’s Hospital, Zhejiang University School of Medicine, No. 261, Huansha Road, Hangzhou, 310006 China; 3 Department of Radiology, Hangzhou Tumor Hospital, Hangzhou, 310006 China; 4 Department of Radiology, Affiliated Hangzhou First People’s Hospital, Zhejiang University School of Medicine, Hangzhou, 310006 China

**Keywords:** Liver cancer, Epstein-barr virus, Cholangiocarcinoma, Case report

## Abstract

The clinical data of a patient with Epstein-barr virus (EBV) associated with cholangiocarcinoma was reported in this paper: a case of a 36-year-old female presented with abdominal pain and systemic skin yellowing combined with skin itching. Laboratory studies showed increase in alanine aminotransferase 242 U/L, aspartate aminotransferase 404 U/L, r-glutamyltransferase 1516 U/L, total bilirubin 308.2 µmol/L and CA199 (101.0 U/ml). AFP (4.5 ng/ml) was normal. CT revealed multiple space-occupying lesions in the liver. PET-CT revealed liver malignant tumor and lymph node metastasis. Liver puncture pathology revealed infiltrative growth of significant heterocyst nests in the liver tissue, which was morphologically consistent with malignant tumors, considering poorly differentiated carcinoma. Pathology suggestion: combining liver puncture with morphology, immunohistochemistry, and EBV in situ hybridization results, it was consistent with EB virus-associated poorly differentiated carcinoma, therefore, consider EBV infection-associated poorly differentiated cholangiocarcinoma (CCA) (LELC morphology). The patient underwent liver transplantation in Hangzhou Shulan Hospital on June 8, 2021 successfully. After surgery, the patient orally took tacrolimus for anti-rejection, entecavir for antiviral therapy, gemcitabine 1.2 g + cis-platinum 30 mg for chemotherapy. After following up for more than 5 months post liver transplantation, the condition of the patient deteriorated. The patient subsequently died. Based on the case of our patient and the review of existing literature, when the patient's serum CA199 increased, AFP did not change significantly, and there was no previous history of hepatitis B. CT revealed a low-density mass in the liver, ring enhancement in the arterial phase, and heterogeneous enhancement of the tumor in the delayed phase. Ring enhancement of the liver lesion mass was observed on MRI. Consider the might possibility of hepatic CCA. When patients showed recurrent tonsillitis at an early age, EBV virus infection should be vigilant and oropharyngeal tissue should persist, diagnosis of EBV-associated liver cancer should be considered. In particular, EBV infection-related liver cancer is relatively rare, the clinician should improve the recognition of the disease to strive for early diagnosis and therapy.

## Background

Primary liver malignancies including hepatocellular carcinoma (HCC) is the most common type of liver cancer and cholangiocarcinoma. Primary liver cancers are a major cause of mortality worldwide and the third leading cause of cancer-related deaths globally [1]. For chemotherapy and radiation therapy are generally invalid, hepatic resection and liver transplantation are the standard of treatment for liver cancers [2]. However, literatures on the Epstein–Barr virus (EBV) cholangiocarcinoma have rarely been reported.

In 1964, Epstein-Barr first discovered human B lymphocytic herpesvirus with herpesvirus particles in malignant lymphocytoma tissues of African children, and established a lymphoblastoid cell line that can be transmitted in biopsies. It is named Epstein-Barr virus (EBV) because of its different characteristics from other members of the known human herpesvirus family [3]. At present, it is known that EBV infection is not only closely related to nasopharyngeal carcinoma, Burkitt's lymphoma, Hodgkin's disease, and human immunodeficiency virus, but also related to the occurrence and development of some foregut-derived organs such as stomach, parotid gland, lung, and thymic carcinoma [4]. Is the development of tumors in the biliary tract, as a foregut-derived organ, associated with EBV infection? EBV has a high infection rate in the population and can remain latent in the human body for life after infection. It can sometimes cause lytic death of infected cells and may also lead to malignant transformation of infected host cells and finally tumorigenesis [5]. Wang Xuemin et al concluded that there is latent infection of EBv in some cholangiocarcinoma cells, especially poorly differentiated cancer cells, suggesting that there are some correlation between EBV infection and the occurrence and development of the cholangiocarcinoma [6].

Undifferentiated carcinomas possessing dense lymphoplasmacytoid stroma are usually classified as lymphoepithelioid carcinoma (LELC) [7]. Among them, nasopharyngeal carcinoma (NPC) is the most common LELC tumor. LELC can also originate from tissues such as salivary glands, stomach, lung, and most of which are associated with EBV [8]. Pathologically, because Lymphoepithelioma-like cholangiocarcinoma (LELCC) is a rare primary adenocarcinoma, LELCC is considered to be an intrahepatic cholangiocarcinoma variant, with only 16 relevant references and 62.5% of cases associated with EBV [9–18]. The following two aspects must be met for its pathological diagnosis: the presence of EBV in glandular tissue, and no abnormal phenotype in lymphoid tissue [19]. Its etiology may be associated with DNA demethylation in tumor tissue [20, 21]. Fukayama M et al reported that many EBV-associated tumor tissues showed DNA demethylation [22, 23]. Some scholars believed that LELC-type cholangiocarcinoma had a better prognosis than conventional type of cholangiocarcinoma [14]. While some scholars believed that gastric and pulmonary LELC tumors had a better prognosis [24, 25]. However, Iezzoni et al concluded that organ types and existence/inexistence of EBV infection had no significance for assessing the prognosis of LELC tumors [8].

An unusual case of cholangiocarcinoma with a lymphoepithelioma-like appearance associated with EBV infection with poor prognosis was reported. Although previous studies have reported the EBV cholangiocarcinoma pathologic features, few studies have revealed the clinic features, laboratory tests, imaging characteristics and clinical course and outcomes. This study aims to highlight the clinical and imaging characteristic, pathologic features, the treatment as well as the outcome of the patient. Furthermore, a review of the literature was conducted in the present study to improve the recognition of the disease, and finally to strive for early diagnosis and therapy.

## Case presentation

A 36-year-old female patient was admitted to the Department of Hepatobiliary Surgery, Affiliated Hangzhou First People's Hospital, Zhejiang University School of Medicine on April 28, 2021 with complaints of middle and upper abdominal pain and discomfort for more than 1 month combined with systemic skin yellowing with skin itching for 2 weeks. One month ago, the patient had middle and upper abdominal pain and discomfort without obvious inducement, which was intolerable. She visited Zhongshan Hospital, Fudan University. CA199:101.0 U/ml, AFP: 4.5 ng/ml. Abdominal CT showed multiple space-occupying lesions in the liver, PET-CT showed multiple MTs, hilar lymph node metastasis in the liver, and cervical lymphadenitis. Liver mass puncture was performed, On April 7, 2021, pathology report showed: consider poorly differentiated carcinoma, and it is recommended to consider primary poorly differentiated carcinoma of the liver after clinical exclusion of metastasis. Immunohistochemistry: Hepa (partially weak+), ARG-1 (−), CK7 (−), CK19 (+), CK20 (−), CDX2 (bits of weak +), p63 (−), p40 (−), SATB2 (weak+), Ki-37 (80% positive), Syn (−), CgA (−), CD56 (−), GATA3 (weak+), PAX8 (−), TTF-1 (−), NapsinA (−), a-Inhibin (−), S-100 (−), SF1 (+), ER (−), PR (−), CK {pan} (+), CK8 (+), SALL4 (individual weak +), CD30 (−), PAX-5 (−), SMA (−), Des (weak +), HMB-45 (−), AFP (−), LCA (lymphocyte +), OCT-4 (−), PLAP (−), CD34 (−). The patient did not receive further treatment. Two weeks ago, the patient developed gradually aggravated abdominal pain, yellow sclera and yellowish skin all over the body, with skin itching. The patient visited to our department for further treatment, and was admitted to the outpatient department for liver malignant tumor. The patient had a history of diabetes for 1 year and was regularly treated with 1 tablet of metformin (Tid) and 1 tablet of acarbose (Tid) orally. Physical examination on admission: right upper quadrant tenderness +, no rebound tenderness, no enclosed mass, negative Murphy's sign, and negative shifting dullness.

**Fig. 1:**
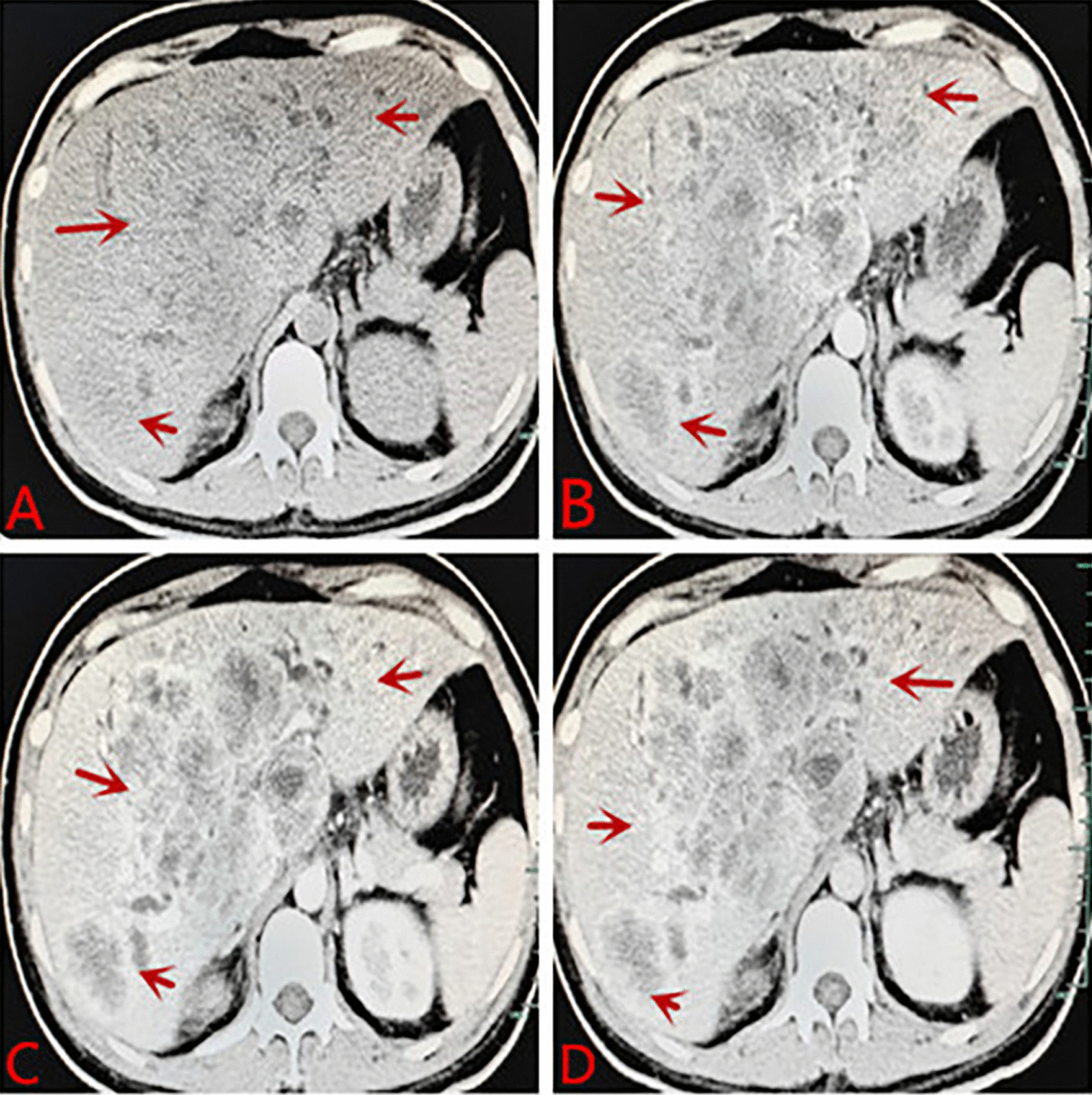
**A** Plane CT scan indicates less regular shape of liver, diffuse mass-like and nodular slightly hypodense lesions, some of which were fused. **B** Contrast-enhanced CT: significant ring enhancement in the arterial phase. **C**, **D** Continuous ring enhancement in the venous and delayed phases, local wall thinning and irritability in the right and left branches of the portal vein. Consider malignant tumors, a high possibility of hepatocellular carcinoma or cholangiocarcinoma, with local invasion of the right and left branches of the portal vein, hilar lymphadenopathy, and a small amount of ascites

**Fig. 2:**
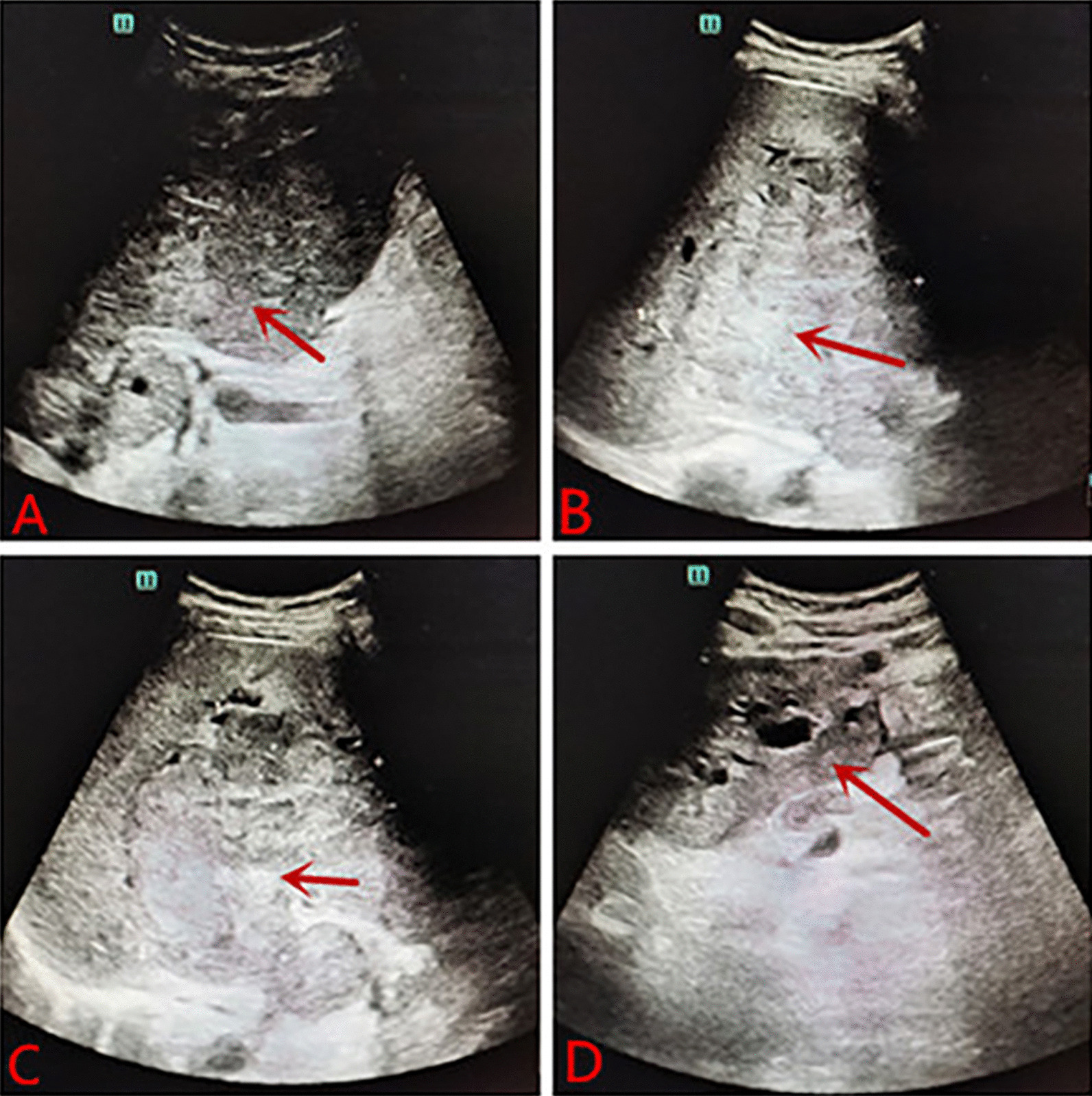
**A** Abdominal ultrasound: size increased liver, and multiple hypoechoic masses observed. **B**–**D** Multiple hypoechoic masses in liver, the large intrahepatic mass is about 11.5 * 10.8 * 10.2 cm, located at the right, consider the possibility of liver cancer

**Fig. 3:**
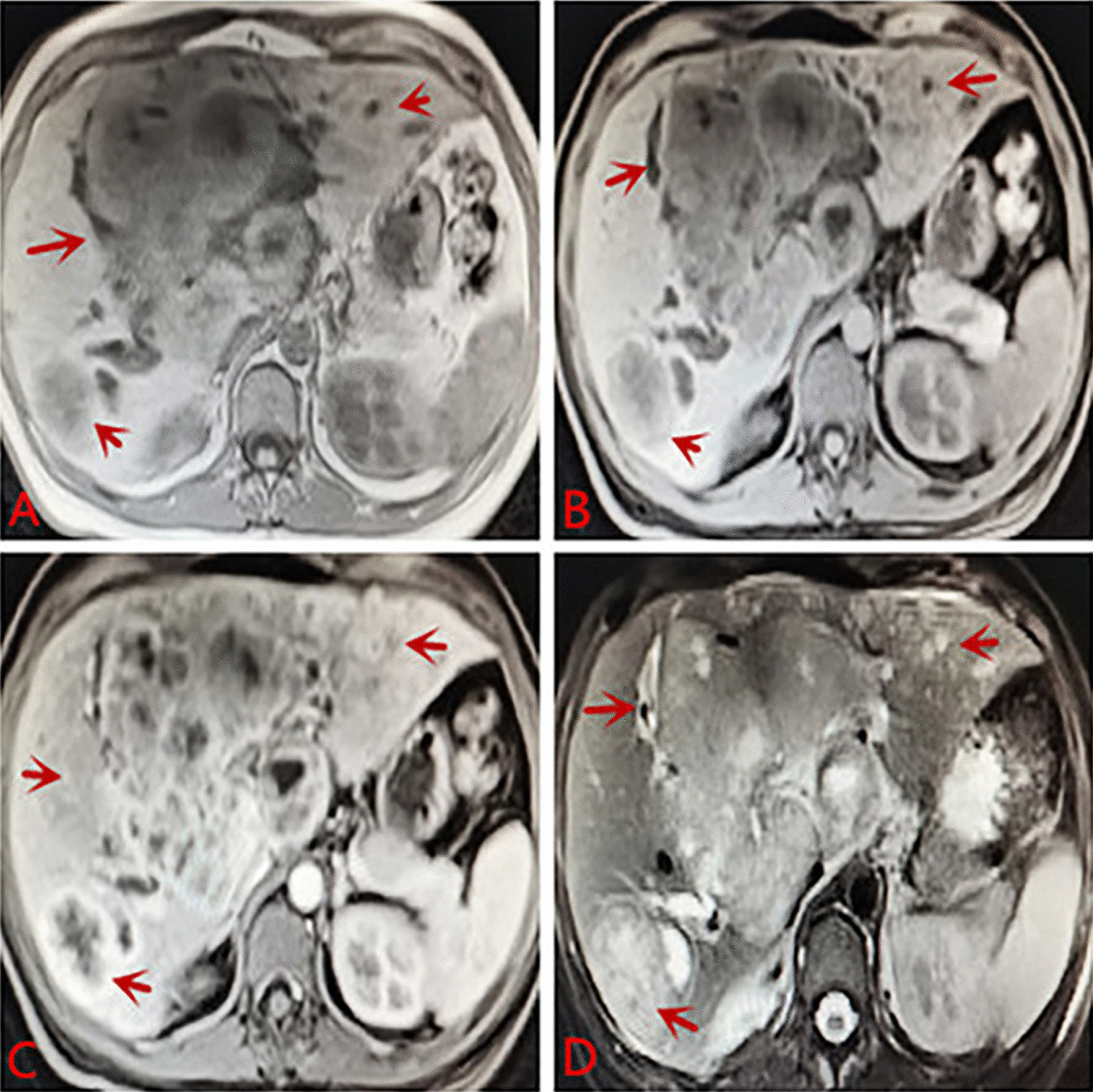
**A** Plain MRI scan: increased liver volume with multiple nodules and mass shadows inside, showing slightly long T1 and slightly long T2 signals, liquefaction necrosis changes were observed in the center of the lesion, and some lesions appeared to be fused. **B**–**D** Contrast-enhanced scans showed mild enhancement of nodular mass shadows and ring-like enhancement changes. Consider malignant tumor combine with intrahepatic multiple metastases. The left branch of the portal vein was not clearly displayed, consider possible involvement. The right branch of the portal vein was suspect been locally invaded, the hepatic vein and the bile duct in the hilar area were not clearly displayed, the bile duct at the upper level was slightly dilated, the left upper quadrant was turbid, consider the possibility of local nodules

Laboratory tests after admission: liver function: alanine aminotransferase 242 U/L, aspartate aminotransferase 404 U/L, r-glutamyltransferase 1516 U/L, total protein 63.4 g/L, albumin 27.6 g/L, globulin 35.8 g/L, total bilirubin 308.2 µmol/L, conjugated bilirubin 224.9 µmol/L, indirect bilirubin 83.3 µmol/L, lactate dehydrogenase 3433 U/L, C-reactive protein 23.5 mg/L, and HbA1c glycosylated hemoglobin 6.4%. Blood coagulation routine: prothrombin time 15.1 s, international normalized ratio 1.42, D-dimer 350.0 µg/L. Blood routine: WBC: 12.7 * 10^9^/L, NEU: 81.9%, hemoglobin: 89 g/L, RBC: 3.48 * 10^12^/L, PLT: 593 * 10^9^/L. Chest CT: pulmonary nodules, exudation in both lower lobes, bilateral pleural reactions, a small amount of right pleural effusion. Contrast-enhanced CT of the abdomen: diffuse mass-like and nodular slightly hypodense lesions, some of which were fused, with significant ring enhancement in the arterial phase, continuous ring enhancement in the venous and delayed phases, and local wall thinning and irritability in the right and left branches of the portal vein. The hepatobiliary ducts were dilated, and the gallbladder wall was thickened and edematous. Malignant tumors were considered, with a high possibility of hepatocellular carcinoma or cholangiocarcinoma, with local invasion of the right and left branches of the portal vein, hilar lymphadenopathy, and a small amount of ascites (see Fig. 1). Abdominal ultrasound: multiple hypoechoic masses in liver, possibility of liver cancer, irritable gallbladder wall, hypoechoic nodules in front of the pancreas, considering enlarged lymph nodes (see Fig. 2). Contrast-enhanced MR of the liver: diffuse nodular mass shadows in the liver, and contrast-enhanced scans showed mild enhancement of nodular mass shadows and ring-like enhancement changes. Consider malignant tumor combine with intrahepatic multiple metastases, the left branch of the portal vein was not clearly displayed, consider possible involvement; the right branch of the portal vein was suspect been locally invaded, the hepatic vein and the bile duct in the hilar area were not clearly displayed, the bile duct at the upper level was slightly dilated, the left upper quadrant was turbid, with the possibility of local nodules (see Fig. 3).

**Fig. 4:**
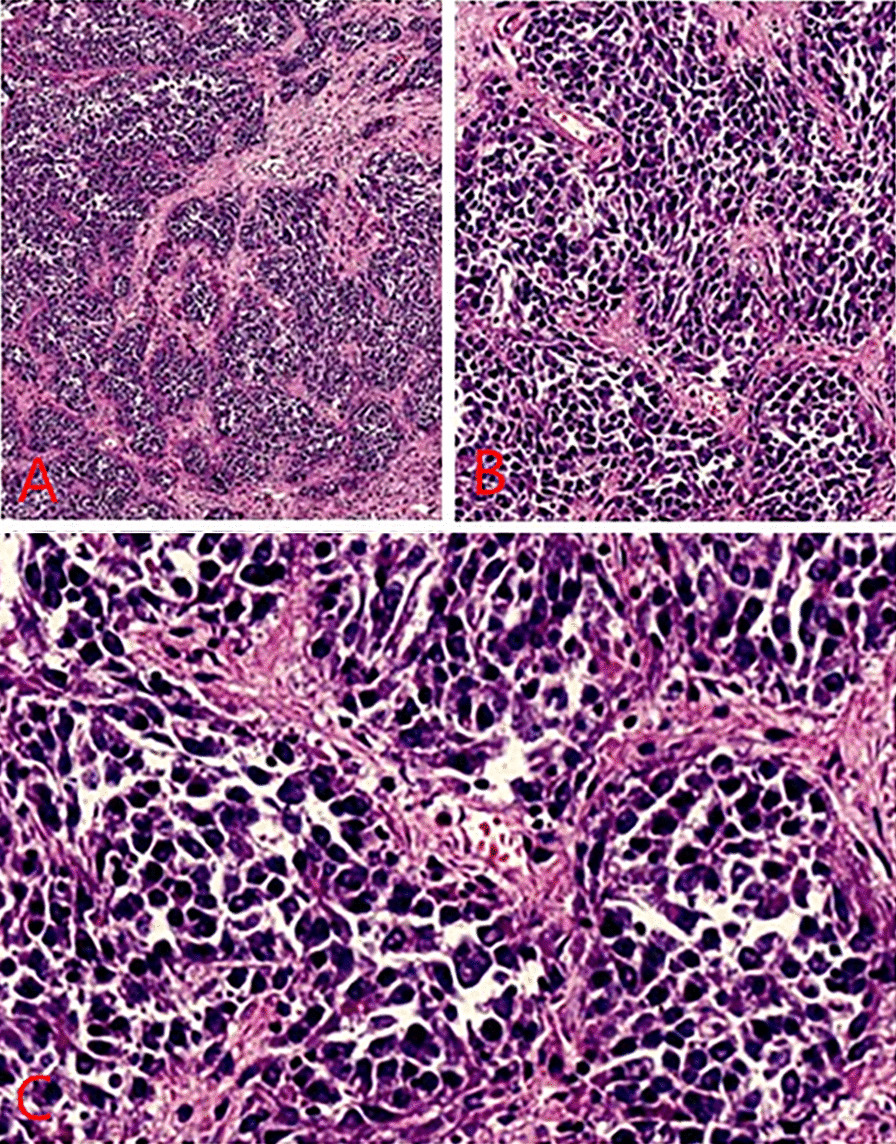
**A** Lymphoepithelioma-like carcinomas (staining, H&E; magnification, × 40). **B** High-power view of the same portion (staining, H&E; magnification, × 100). **C** Further high-power view revealed infiltrative growth of significant heterocyst nests in the liver tissue, which was morphologically consistent with malignant tumors, consider poorly differentiated carcinoma. (staining, H&E; magnification, × 200)

**Fig. 5:**
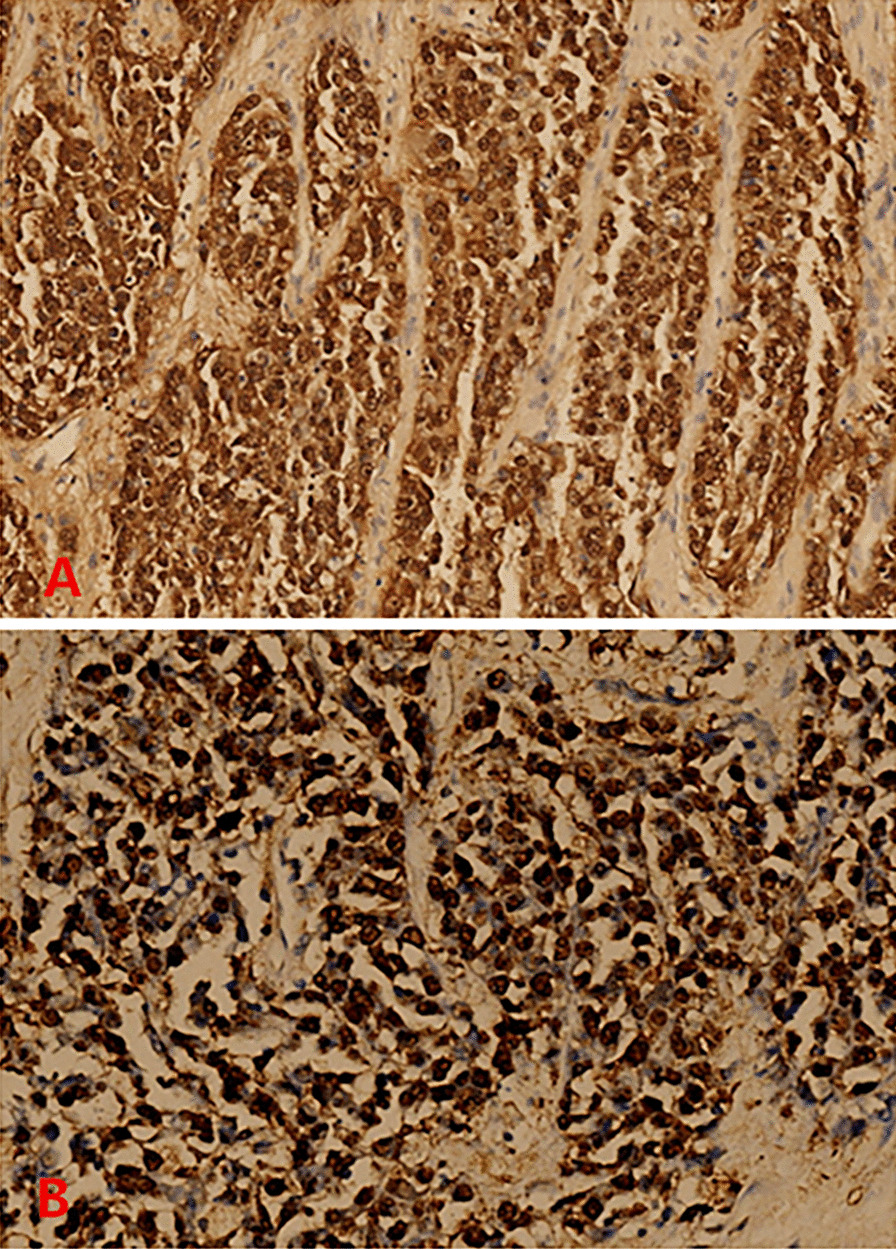
**A** Immunohistochemical results: CK7 foci (+), CK18 (weak + - +), CK19 (weak + - +), CK20 (−), CDX_2_ (weak + - +), AFP (−), Hepar1 (−), Arg-1 (−), GATA-3 (−), Vim (−), P40 a little (+), CD117 (−), CerbB2 (−), Ki-67 (+) 80–90%, EBV in situ hybridization: tumor cells EBER (+). Combining liver puncture with morphology, immunohistochemistry, and EBV in situ hybridization results, it was consistent with EB virus-associated poorly differentiated carcinoma (magnification, × 100). **B** Consider EBV infection-associated poorly differentiated cholangiocarcinoma (LELC morphology) magnification, × 200)

The patient was given ademetionine 1000 mg Qd for choleresis treatment, vitamin K1iInjection 30 mg Qd for thromboprophylaxis, cefoperazone sodium and sulbactam sodium injection 2g Q8h for anti-infection. Transfusion of type O RH positive suspended red blood cells was given to repair anemia and coagulation abnormalities. On May 1, 2021, ultrasound-guided biliary drainage was performed for jaundice reduction treatment, and the right bile duct PTCD conditions were immature. After surgery, the PTCD drainage tube was patent, and the icteric skin and sclera gradually improved. Liver puncture pathology revealed infiltrative growth of significant heterocyst nests in the liver tissue, which was morphologically consistent with malignant tumors, and poorly differentiated carcinoma was considered. Pathology suggestion: combining liver puncture with morphology, immunohistochemistry, and EBV in situ hybridization results, it was consistent with EB virus-associated poorly differentiated carcinoma (See Fig. 4). Consider EBV infection-associated poorly differentiated cholangiocarcinoma (LELC morphology). Immunohistochemical results: CK7 foci (+), CK18 (weak + - +), CK19 (weak + - +), CK20 (−), CDX_2_ (weak + - +), AFP (−), Hepar1 (−), Arg-1 (−), GATA-3 (−), Vim (-), P40 a little (+), CD117 (−), CerbB2 (−), Ki-67 (+) 80%-90%, EBV in situ hybridization: tumor cells EBER (+) (Fig. 5).

The Department of Infectious Diseases was invited for consultation, and the patient's pathology suggested EBV-related tumor. Upon inquiry of medical history, the patient often had fever from an early age, accompanied with tonsillitis and pus moss. The fever could be controlled after the use of antipyretics. The tonsils had been removed in 2015. In summary, EBV infection could be diagnosed. The patient was given intravenous drip of phosphopotassium sodium chloride injection (tolerable) 3g Q12h for antiviral symptomatic treatment. He was transferred to Shulan Hospital for liver transplantation. The patient underwent liver transplantation in Hangzhou Shulan Hospital on June 8, 2021, and the operation was successful. The pathology revealed EBV-related poorly differentiated cholangiocarcinoma. Regional lymph nodes: (groups 7, 8 and 9) 7/8 lymph node cancer metastases. After surgery, the patient orally took tacrolimus for anti-rejection, entecavir for antiviral therapy, gemcitabine 1.2 g combined with cis-platinum 30 mg for chemotherapy. With followed up for more than 5 months after liver transplantation, and the condition of the patient deteriorated. The patient is in critical condition currently, and the patient's family had given up treatment. The patient subsequently died. See Table 1 for patient condition and treatment process, and abbreviations

**Table 1:** Test results and patient symptoms time

Time/site	Clinical features	Laboratory tests	Imageological examinations	Pathology	Treatment
On March 2021; Zhongshan Hospital, Fudan University	Middle and upper abdominal pain	CA199: 101.0U/ml, AFP: 4.5ng/ml	CT: multiple space-occupying lesions in the liver PET-CT: multiple MTs, hilar lymph node metastasis in the liver	Pathology: poorly differentiated carcinoma of the liver	
On April 2021 Hangzhou First People's Hospital	Intolerable abdominal pain, systemic skin yellowing with skin itching	ALT:242U/L AST: 404 U/L Total bilirubin 308.2 µmol/L Prothrombin time: 15.1 s Blood routine: WBC: 12.7 * 10^9^/L, hemoglobin: 89 g/L, RBC: 3.48 * 10^12^/L Hepatitis B surface antigen negative	CT: Malignant tumors were considered, with a high possibility of hepatocellular carcinoma or cholangiocarcinoma MRI: consider malignant tumor combine with intrahepatic multiple metastases Abdominal ultrasound: multiple hypoechoic masses in liver, possibility of liver cancer	Pathology and immunohistochemistry: EBV infection-associated poorly differentiated cholangiocarcinoma	1. Ademetionine 1000 mg Qd for choleresis treatment 2. Vitamin K1 injection 30 mg Qd for thromboprophylaxis, cefoperazone 3. Sodium and sulbactam sodium injection 2g Q8h for anti-infection 4. Transfusion of type O RH positive suspended red blood cells 5. Ultrasound-guided biliary drainage for jaundice reduction treatment
On June 2021 Hangzhou Shulan Hospital	Abdominal pain			EBV infection-associated poorly differentiated cholangiocarcinoma. Regional lymph nodes: (groups 7, 8 and 9) 7/8 lymph node cancer metastases	1. Liver transplantation 2. Tacrolimus for anti-rejection 3. Entecavir for antiviral therapy 4. Gemcitabine 1.2 g combined with cis-platinum 30 mg for chemotherapy
On July 2021 Hangzhou First People's Hospital	Status post liver transplant, low-grade fever	Bile bacterial culture: enterococcus faecalis	Abdominal ultrasound: right pleural effusion, peritoneal effusion		Piperacillin tazobactam 4.5g Q8h Intravenous drip for anti-infection
On December 2021 At home	The condition deteriorated				Give up treatment The patient died

## Discussion

Cholangiocarcinoma (CCA) is a malignant tumor arising from the biliary epithelium and its surrounding glands and is relatively rare, however, EB virus-associated CCA is even rarer and its prognosis is extremely poor. For special types of CCA, if the diagnosis is made early, early surgical treatment or combination with systemic chemotherapy and other regimens may delay the development of the disease and prolong survival. WAKIZAKA K et al believed that the laboratory and imaging features of CCA were: elevated serum CA199, insignificant elevation of AFP, no previous history of hepatitis B, a low-density mass in the liver by CT, ring enhancement in the arterial phase, and heterogeneous enhancement of the tumor in the delayed phase. Ring enhancement of the liver lesion mass was observed on MRI [26]. In this case, the patient's clinical symptom was abdominal pain, and the laboratory test results revealed CA199:101.0 U/mL and AFP: 4.5 ng/mL. There was no history of hepatitis B. Contrast-enhanced CT of the abdomen revealed diffuse mass-like and nodular slightly hypodense lesions with significant ring enhancement in the arterial phase and continuous ring enhancement in the venous and delayed phases, showing typical imaging findings of intrahepatic cholangiocarcinoma, which was consistent with previous references. MRI examination of the liver can be used to assess the infiltration of the tumor along the extension of the biliary tract. Contrast-enhanced MRI of the liver in this patient revealed mild enhancement of diffuse nodular mass shadows and ring-like enhancement changes on contrast-enhanced scans. It conforms to previous references.

EBV is a herpes virus and a widespread DNA virus. After individuals are infected with EBV at an early age, the virus often persists in oropharyngeal tissue, cervical epithelium and lymphocytes. Malignant tumors associated with EBV infection mainly include nasopharyngeal carcinoma, lymphoma and gastric cancer [27]. However, whether there is an association between EBV infection and hepatocellular carcinoma development has rarely been reported. Randbawa PS et al have reported the presence of EBV in the recipient liver of liver transplantation. EBER-1 is the RNA encoded by EBV during the latent infection period. The authors used in situ hybridization to detect EBER-1 in liver cancer tissues, suggesting that EBV invasion also occurs in the liver. Since EBER-1 is not found in the liver tissues, including cirrhotic tissues and atypical hyperplastic hepatocytes, it is considered that EBV infection occurs after hepatocyte carcinogenesis [28]. Howe JG et al believed that EBV in HCC cells could promote cell proliferation, and EBER-1 positive cases were seen in poorly differentiated hepatocellular carcinoma, but dysplasia hepatocytes were negative, so it was concluded that EBER-1 promoted the progression of HCC, and HCC associated with EBV infection might be expanded from an EBV-infected cell clone [29]. Domestic scholars have reported that with the progression of HBV-related liver diseases (including hepatitis B, hepatitis B cirrhosis and liver cancer), the positive rate of EB virus antibody is significantly increased, and EB virus antibody positive patients have a poor prognosis and a high mortality rate [30]. Xuemin Wang et al., some Chinese scholars, has reported that EBV infection has certain correlation with the occurrence and development of CCA. It is believed that the positive expression rate of LMP-1 is related to the differentiation degree of CCA cells. The lower the differentiation degree, the higher the positive expression rate. It is speculated that poorly differentiated carcinoma allows EBV amplification in poorly differentiated carcinoma of the bile duct. The expanded EBV can endow the growth advantage of the tumor and promote the proliferation of cancer cells, resulting in the invasion and development of the tumor [6]. Pathological findings in this case suggested that EBV infection-associated poorly differentiated cholangiocarcinoma (LELCs) was poorly differentiated hepatocellular carcinoma, and the patient often had fever, tonsillitis, and pus moss at an early age. The tonsils were removed in 2015. In summary, it could be diagnosed as EBV infection. The prognosis of this patient was poor, and the condition of this patient was consistent with previous reports in the literature.

In this case, although the pathology suggested EBV infection-associated poorly differentiated cholangiocarcinoma (LELC), the pathological section of the patient showed less lymphocyte infiltration in the tumor background, and the tumor cells showed diffuse and consistent expression of EBER. Hsu et al have reported the pathology of the first patient with undifferentiated LELC, and it may be difficult to diagnose CCA complicated by LELC type due to the heterogeneity of tumor components [7]. Labgaa I et al believed that LELC was usually associated with EBV infection, studied showed that, in 28 cases of lymphoepithelioma-like CCA patients, 92% were Asian, and the 5-year survival of patients with lymphoepithelioma-like CCA was longer than that of patients with simple CCA (100% vs. 13.2%) [31]. Austin Lin et al have reported that two patients with intrahepatic CCA of LELC (African descent), could not undergo surgery due to tumor progression, and had a poor prognosis [32]. In this case, the patient was young, and although liver transplantation, systemic chemotherapy and other tumors were performed, the progression was rapid and the prognosis was still poor. It is consistent with the report of Austin Lin et al.

Because the rarity of special types of CCA cases, there is no uniform regimen for the treatment of this type of disease, and the prognosis varies among treatment regimens. O'Connor et al concluded that special types of CCA should be actively treated through surgery and chemotherapy [33]. Nakhoul et al. believed that antiviral therapy was required for the treatment of hepatic EBV-associated tumors, and the commonly used antiviral drugs were ganciclovir and acyclovir [4]. Austin Lin et al believed that FOLFOX chemotherapy regimen could be given to patients who could not undergo surgical treatment [32]. Labgaa et al. believed that (PDL-1) inhibitors was available for nasopharyngeal carcinoma treatment, so they were also effective for patients with EB virus-associated LELCCCA. In this case, the patient's treatment regimen was: liver transplantation, entecavir for antiviral therapy, gemcitabine 1.2 g combined with cis-platinum 30 mg for chemotherapy. After more than 5 months of follow-up after liver transplantation, the patient died with deteriorated condition. Combined with the clinical data of this patient, EBV infection-related CCA has a poor prognosis and a high mortality rate.

## Conclusion

In summary, when the patient's serum CA199 increased, AFP did not change significantly, and there was no previous history of hepatitis B. CT revealed a low-density mass in the liver, ring enhancement in the arterial phase, and heterogeneous enhancement of the tumor in the delayed phase. Ring enhancement of the liver lesion mass was observed on MRI. The possibility of hepatic CCA should be considered. When patients have recurrent tonsillitis at an early age, EBV virus infection should be vigilant and oropharyngeal tissue should persist, EBV in liver cancer cells has a role in promoting cell proliferation, and the prognosis is often poor. For patients with suspected CCA, EB virus immunohistochemical detection is recommended during liver biopsy for early detection and treatment. Antiviral treatment can be combined with antiviral surgery, as well as chemotherapy when necessary. The mechanism of infection of CCA by EBV is unknown. Considering the small number of cases, this conclusion needs to be confirmed by further studies.

## Data Availability

All data generated or analyzed during this study are included in this published article.

## Abbreviations

PET: Positron emission tomography
AST: Aspartate aminotransferase
ALT: Alanine aminotransferase
CA 19-9: Carbohydrate antigen 19-9
MRI: Magnetic resonance imaging
LELC: Lymphoepithelioma-like carcinomas
LELCC: Lymphoepithelioma-like cholangiocarcinoma
HCC: Hepatocellular carcinoma
EBV: Epstein–Barr virus
CCA: Cholangiocarcinoma
